# R-AI-diographers: a European survey on perceived impact of AI on professional identity, careers, and radiographers’ roles

**DOI:** 10.1186/s13244-025-01918-6

**Published:** 2025-02-17

**Authors:** Nikolaos Stogiannos, Gemma Walsh, Benard Ohene-Botwe, Kevin McHugh, Ben Potts, Winnie Tam, Chris O’Sullivan, Anton Sheahan Quinsten, Christopher Gibson, Rodrigo Garcia Gorga, David Sipos, Elona Dybeli, Moreno Zanardo, Cláudia Sá dos Reis, Nejc Mekis, Carst Buissink, Andrew England, Charlotte Beardmore, Altino Cunha, Amanda Goodall, Janice St John-Matthews, Mark McEntee, Yiannis Kyratsis, Christina Malamateniou

**Affiliations:** 1 Department of Midwifery & Radiography, City St George’s, University of London, London, UK; 2 Magnitiki Tomografia Kerkyras, Kerkira, Greece; 3 School of Dental, Health and Care Professions, University of Portsmouth, Portsmith, UK; 4 University Hospital Southampton NHS Foundation Trust, Southampton, UK; 5 Institute of Diagnostic and Interventional Radiology and Neuroradiology, University Hospital Essen, Essen, Germany; 6 German Society of Medical Technologists for Radiology, Berlin, Germany; 7 Maidstone and Tunbridge Wells NHS Trust, Tunbridge Wells, UK; 8 Nuclear Medicine Service, Hospital Universitari Parc Taulí, Sabadell, Spain; 9 Department of Medical Imaging, Faculty of Health Sciences, University of Pécs, Pécs, Hungary; 10 Department of Medical Technical Specialties, Faculty of Medical Technical Sciences, University of Elbasan “Aleksander Xhuvani”, Elbasan, Albania; 11 Unit of Radiology, IRCCS Policlinico San Donato, San Donato Milanese, Italy; 12 School of Health Sciences (HESAV), University of Applied Sciences and Arts Western Switzerland (HES-SO), Lausanne, Switzerland; 13 Faculty of Health Sciences, University of Ljubljana, Ljubljana, Slovenia; 14 European Federation of Radiographer Societies, Cumiera, Portugal; 15 Hanze University of Applied Sciences, Groningen, The Netherlands; 16 Discipline of Medical Imaging and Radiation Therapy, University College Cork, Cork, Ireland; 17 The Society and College of Radiographers, London, UK; 18 Bayes Business School, City St George’s, University of London, London, UK; 19 Office of the Chief Allied Health Professions Office (CAHPO), National Health Service (NHS) England, London, UK; 20 Health Services Management & Organisation (HSMO), Erasmus School of Health Policy & Management, Erasmus University Rotterdam, Rotterdam, The Netherlands; 21 European Society of Medical Imaging Informatics, Vienna, Austria; 22 Department of Neuroimaging, King’s College London, London, UK

**Keywords:** Artificial intelligence, Radiographers, Europe, Professional identity, Impact

## Abstract

**Objectives:**

Radiographers use advanced medical imaging and radiotherapy (MIRT) equipment. They are also a digitally mature and digitally resilient workforce in healthcare. Artificial intelligence is already changing their clinical practice and roles in data acquisition, post-processing, and workflow management. It is therefore vital to understand the impact of AI on the careers, roles and professional identity of radiographers, as key stakeholders of the digital transformation of healthcare within the medical imaging ecosystem.

**Methods:**

A European radiographer survey, endorsed by the European Federation of Radiographer Societies (EFRS), was distributed online. It was piloted with twelve radiographers and translated into eight languages. Although this study included both qualitative and quantitative results, this paper emphasises the quantitative aspect.

**Results:**

A total of 2206 European radiographers have responded from 37 different countries. Despite some concerns around workforce deskilling, future professional identity, and job prospects, participants showed overall optimistic views about the use of AI in healthcare. This was particularly strong for those with prior AI education (mean: 2.15 vs. 1.89; *p*-value: < 0.001), hands-on experience with AI (correlation: 0.047; *p*-value: 0.038), from countries with higher digital literacy (mean: 2.00 vs.1.93; *p*-value: 0.027) and a higher academic level of radiography education (mean: 3.28 vs. 3.15; *p*-value: 0.002). Men appeared slightly more enthused about the development of technological skills and women about the honing of patient-centred care skills. Finally, interprofessional collaboration was seen as essential not only for the seamless clinical integration of AI but also for supporting patient benefit.

**Conclusion:**

While AI implementation advances, AI education needs to keep at pace to ensure acceptability, trust, and safe use of this technology by healthcare professionals, minimising their concerns around professional role changes and enabling them to see the opportunities of service transformation.

**Critical relevance statement:**

This paper aims to map out the perceived impact of AI on the professional identity and careers of European radiographers.

**Key Points:**

- AI is impacting radiographers’ clinical practice and changing their professional identity.
- Despite increasing AI awareness, AI education is still lacking across Europe.
- AI education is key for AI acceptability and trust by radiographers, which facilitates AI implementation and service transformation.

**Graphical Abstract:**

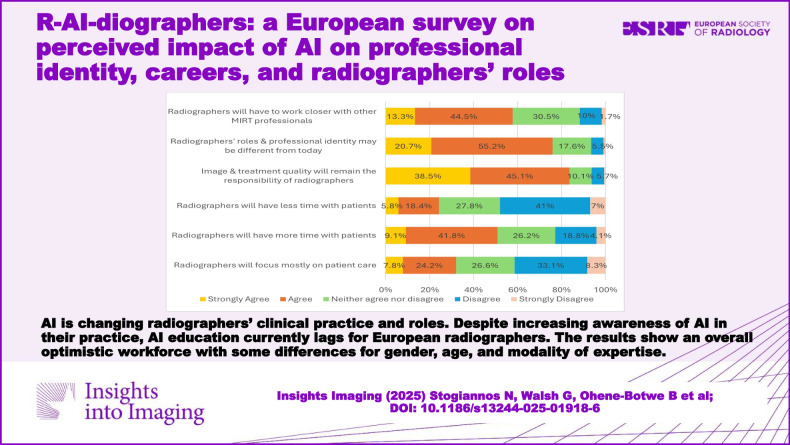

## Introduction

AI has been deployed in different aspects of radiography clinical practice [1], such as data acquisition and data analysis, but has also shown promise in mitigating diagnostic or data acquisition errors, streamlining image analysis, and optimising workflows [1, 2]. Radiographers, who are expert professionals working in medical imaging and radiotherapy (MIRT), are already working closely with AI tools. Despite the digital resilience of radiography professionals, carved through years of adaptation to new technologies, there is considerable concern that AI might be more disruptive; it is expected that AI integration will significantly impact career pathways, professional identity and roles of radiographers [3].

Professional identity can be defined as ‘the way that professionals see themselves in terms of who they are and what they do’ [4]. Professional identity is socially constructed, and it is shaped and evolved through interactions of individuals with ideas, people, cultures, and social groups. Therefore, professional identity entails a socialisation process, through which individuals adopt social norms and values [5]. Professional identity entails a core part of someone’s personal identity. For radiographers, it encompasses both technological competencies and patient care skills, and the duality of radiographers’ professional identity has been well-recognised [6]. In addition, radiographers’ professional identity includes the ethical principles associated with radiography practice, their professionalism, attitude, knowledge of governance, and their role perception in their area of practice, as a component of professional identity [7]. There are newly proposed professional archetypes for healthcare professionals changing roles because of AI, which could form the basis of future professional identities [8].

Different professionals within the MIRT ecosystem, including radiologists, medical physicists, and radiographers, voice concerns about the impact of AI on human skills, competencies, and career prospects [9–12]. A level of apprehension is particularly related to safety and governance for AI clinical implementation and ongoing developments. Previous studies [10, 13, 14] have shown that radiographers have concerns regarding the adoption of AI for future job prospects [10] whilst others [13] propose that new roles should be created to enable radiographers to harness the benefits of AI [14]. However, most studies remain segregated, with a generic overview of the impact of AI and without a large enough sample size to be able to draw more definitive conclusions. Moreover, the above studies have been conducted across a single country only; in addition, none of them has explicitly explored the impact of AI on the professional identity and career of radiographers. Therefore, a literature gap exists in this field.

This study aimed to explore the perceived impact of AI on European radiographers’ careers, roles and professional identity. The objectives of this study are: (1) to provide an overview of AI awareness among European radiographers, (2) to map out their perceptions regarding the impact of AI in radiography practice and responsibilities, (3) to highlight their views on the impact of AI on their roles and professional identity, and (4) to explore the correlation of these findings with key demographic features like digital literacy and level of education.

## Methods

### Study design and reporting

This is a cross-sectional study. The Strengthening the Reporting of Observational Studies in Epidemiology (STROBE) [15] reporting for cross-sectional studies and Checklist for Reporting Results of Internet E-Surveys (CHERRIES) guidelines for e-surveys were used in this work [16].

### Instrument

An online survey was hosted on Qualtrics (Qualtrics, Provo, Utah, USA). Its development was based on prior interviews and focus group discussions involving European experts in the fields of radiography and AI, and a rapid review of the relevant literature [17]. The survey was finalised in content and format after successive discussions of the research team to reach consensus. A summary of the survey structure can be found in the Appendix. Piloting was performed in a diverse group of experts (*n* = 12), from different countries and clinical subspecialties within radiography. The internal consistency of the survey was measured using Cronbach’s alpha coefficient [18]. This indicated that the internal consistency of the survey was within acceptable limits (α = 0.78).

### Project coordination

To increase uptake and ensure a multicultural representation from different countries [19], the survey was translated from English into eight different languages (French, German, Italian, Spanish, Greek, Slovenian, Hungarian, and Albanian). Translation of the survey was conducted by radiography colleagues who were native speakers. The choice of languages represents the five most spoken languages in Europe [20] and the rest was based on the capacity of individual researchers. Forward translation was employed for the survey questions whilst backward translation was employed for analysis [21]. Regular briefings with senior authors ensured the team was fully informed and familiar with the processes, governance, and data management. All queries were addressed during two online meetings with the wider team, to ensure consistency of the work across the different languages [22]. Two experienced research assistants were allocated to the project to ensure smooth coordination and minimisation of errors. The survey included 36 questions. This comprised 12 closed-type questions, 2 multiple-choice questions, and 16 questions that measured specific attitudes of the respondents using a 5-point Likert-type scale [23]. The last part of the survey included six open-ended questions. The respondents were asked to provide basic demographic data, including AI knowledge and experience in the use of AI, prior education on AI, and their perceptions of the impact of AI on the roles, identity, and career prospects of radiographers. It must be noted that, although both qualitative and quantitative data were collected, this paper only reports the quantitative aspects of this survey.

The survey completion time was approximately 15 min. Respondents were able to return to previous survey questions, if required. To increase participation and offer flexibility, responses could also be saved to allow for delayed completion within a 24-h period.

### Participants

Inclusion criteria for participants were: (1) being over 18 years of age; (2) being a radiographer in any modality or role, according to the European Skills, Competences, Qualifications and Occupations (ESCO) [24] classification of the profession (including diagnostic and therapeutic radiographers, sonographers, nuclear medicine technologists, radiation therapists, students, and retired radiographers); (3) working within Europe, including the UK (in clinical settings, academia, research, industry, or professional/regulatory bodies).

### Data collection

The survey was recruiting between June 3 and August 31, 2023. It was distributed through the membership of the European Federation of Radiographer Societies (EFRS) and the researchers’ personal and professional networks and social media. Personalised links were sent via email to prospective participants. This study also recruited at the UK Imaging & Oncology Congress (UKIO) 2023 Research Hub.

### Data analysis

All data was cleared of incomplete entries and then sorted by country. This was necessary for further analysis because some countries, like Switzerland or Luxembourg, have multiple official languages. This enabled the team to explore correlations between acquired data and different countries and extract meaningful results. All data were analysed using descriptive statistics on the Statistical Package for the Social Sciences (SPSS), version 28.0 (IBM Corp, Armonk, New York). Descriptive analysis included measurements of absolute and relative frequencies of distribution, and mean scores where appropriate. Tables and graphs were created to summarise results and visualise key findings. Inferential statistics was also employed to explore relationships between variables. The Kruskal–Wallis test [25] was used to compare perspectives on AI between certain demographic groups. This included gender identity, countries with high (> 56% of individuals with basic digital skills) and low digital literacy (< 56% of individuals with basic digital skills) [26] (Table 1), younger (18–35 years) and older respondents (36+ years), different educational levels (bachelor’s, master’s etc.), and those with different AI education levels. Spearman’s rho tests [25] were performed to assess correlations between certain demographic variables, e.g., experience with AI and years of working experience and opinions about AI integration in radiography. The level of statistical significance was set to *p* < 0.05.

**Table 1:** Low and high digital literacy countries based on data from the European Union [26]

Low digital literacy countries	High digital literacy countries
Bulgaria	Belgium
Germany	Czechia
Greece	Denmark
Italy	Estonia
Cyprus	Ireland
Latvia	Spain
Lithuania	France
Hungary	Croatia
Poland	Luxemburg
Romania	Malta
Slovenia	Netherlands
	Austria
	Portugal
	Slovakia
	Finland
	Sweden
	Iceland
	Norway

Based on that data countries with over 56% of the population having some basic literacy skills are classified as high digital literacy countries

### Ethics

Ethical approval was obtained from City St George’s, University of London School of Health and Psychological Sciences Ethics Committee (ETH2223-1346).

An electronic information sheet and electronic consent were integrated on the introductory page of the online survey [27]. As this was an anonymous survey, data could not be withdrawn. During UKIO 2023, voluntary recruitment of participants was achieved through the availability of a dedicated QR code that could be scanned from printed posters in the conference’s research hub. Participants could freely choose to answer surveys from different available studies from that hub. Once a participant chose to answer our study, they were given access to a private workstation to complete it on their phone and on their own time. Privacy was therefore ensured through anonymous data collection and voluntary survey completion.

## Results

**Fig. 1:**
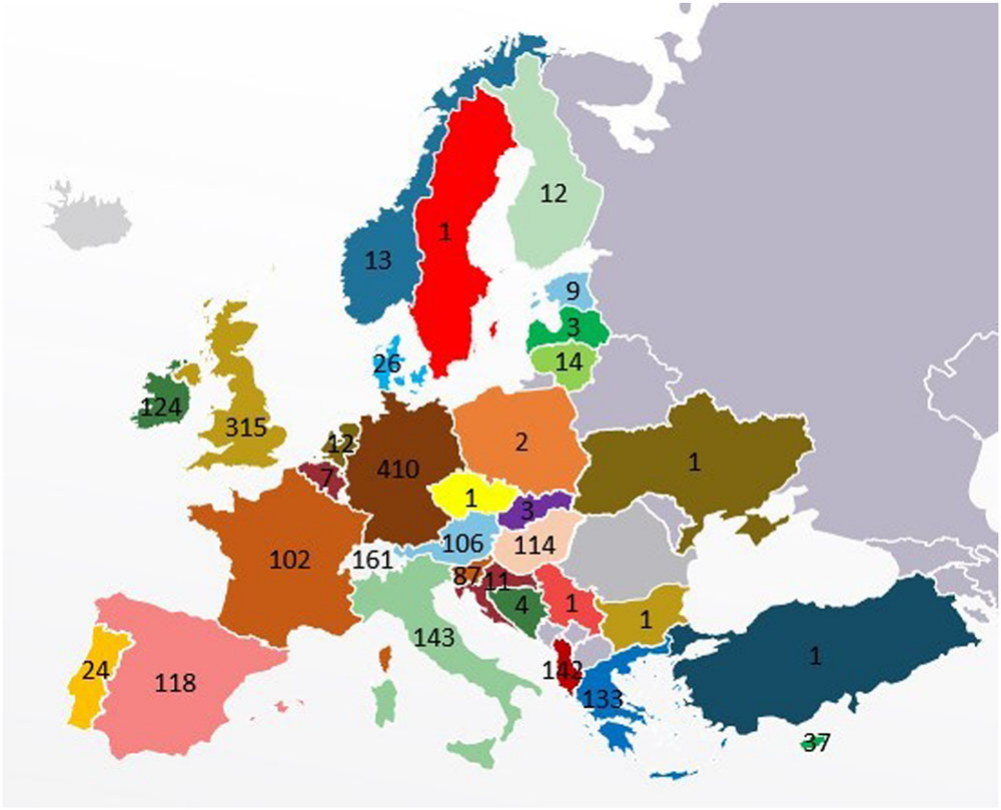
Geographical distribution of the respondents. Relevant frequencies are superimposed on each country

Of the initial 3125 participants who accessed the survey, a total of 2206 valid responses were received. The responses were distributed across 37 European countries (Fig. 1). Due to survey attrition, not all questions were answered by all respondents; hence, all frequencies and percentages refer to the actual number of responses received for each question.

### Basic demographics

Table 2 summarises the basic demographic data of the respondents. The most prevalent group in each category is highlighted in bold.

**Table 2:** Demographic data of the respondents

Gender identity	
Female	**64.0% (*n*** = **1411)**
Male	34.6% (*n* = 763)
Prefer not to say	0.9% (*n* = 19)
Non-binary	0.5% (*n* = 13)
Age	
18–25 years old	17.9% (*n* = 395)
26–35 years old	**28.7% (*n*** = **634)**
36–45 years old	24.8% (*n* = 546)
46–55 years old	18.3% (*n* = 403)
56–65 years old	9.1% (*n* = 202)
> 65 years old	0.9% (*n* = 20)
Prefer not to say	0.3% (*n* = 6)
Years of experience	
0–2 years	11.9% (*n* = 262)
3–5 years	12.7% (*n* = 281)
6–10 years	15.7% (*n* = 346)
11–20 years	25.0% (*n* = 552)
> 20 years	**31.3% (*n*** = **690)**
Not practicing	2.9% (*n* = 64)
Retired	0.5% (*n* = 11)
Radiographic specialty	
Diagnostic radiographer	**59.9% (*n*** = **1320)**
Both diagnostic and therapeutic radiographer (dual qualification)	15.3% (*n* = 338)
Therapeutic radiographer	9.7% (*n* = 215)
Nuclear medicine technologist	7.7% (*n* = 169)
Sonographer	2.1% (*n* = 46)
Other (e.g., all specialties, interventional, administration)	5.3% (*n* = 118)
Highest qualifications	
BSc (or DCR or equivalent)	**32.7% (*n*** = **722)**
Master’s (or MBA or equivalent)	22.6% (*n* = 499)
Postgraduate Diploma	11.5% (*n* = 253)
Undergraduate student	11.1% (*n* = 244)
Postgraduate certificate	5.1% (*n* = 113)
PhD/Professional Doctorate	2.0% (*n* = 44)
Other (e.g., PhD student, vocational training, extended secondary education)	15.0% (*n* = 231)

Most radiographers reported working in public hospitals (*n* = 1335, 61.4%), followed by those working at private hospitals/centres (*n* = 497, 22.5%), research facilities (*n* = 80, 3.6%), and mobile units (*n* = 27, 1.2%). Most respondents (81.5%, *n* = 1798) were practising radiographers, while 17.3% (*n* = 382) of them were undergraduate students, assistant practitioners, or apprentices, and 1.2% (*n* = 26) were retired.

### Knowledge of and experience with AI

**Fig. 2:**
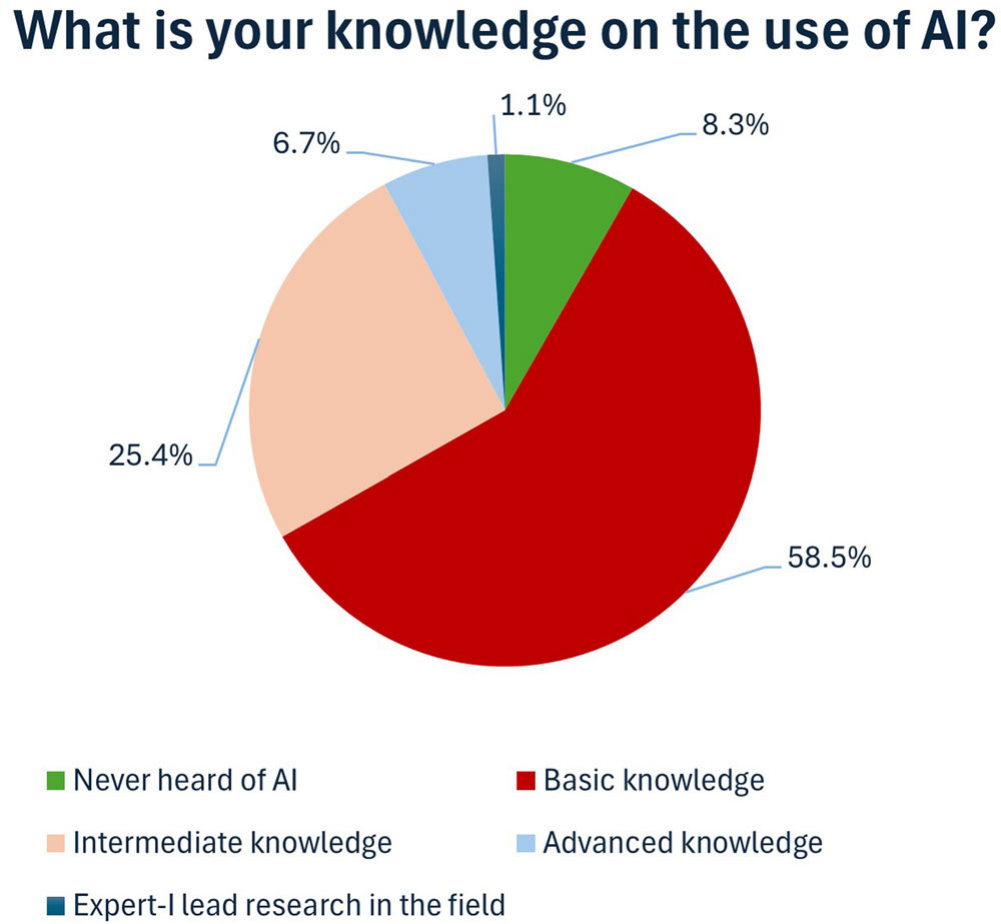
Pie chart illustrating the knowledge of the respondents on the use of AI

Most radiographers reported basic or intermediate knowledge of the use of AI (Fig. 2).

Over half of the respondents (50.5%, *n* = 1114) said that they had never used AI to their knowledge, 27.1% (*n* = 597) reported using AI tools occasionally, 15.0% (*n* = 332) reported daily use of AI in their practice, and 5.4% (*n* = 118) said they were engaged with research/development of AI. The rest (2.0%, *n* = 45) had used AI only once on a trial or demonstration basis, e.g., during exhibitions at conferences.

**Fig. 3:**
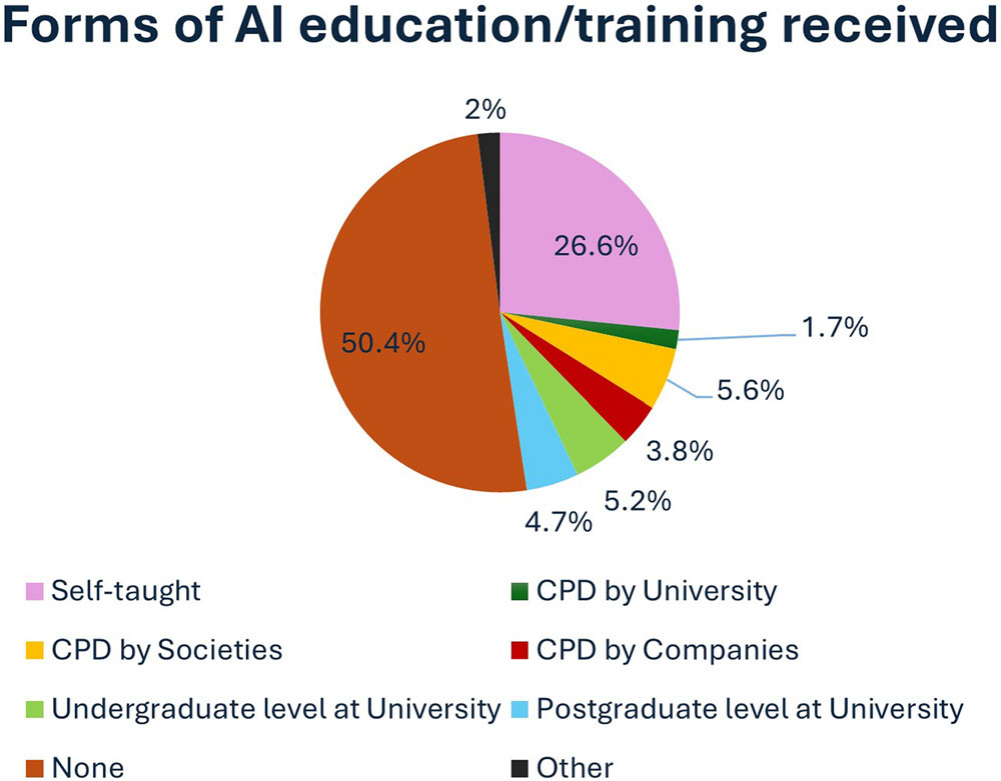
Forms of AI education/training which the respondents had received

More than half (50.4%, *n* = 1113) reported that they had no prior AI education and about a quarter of all respondents were self-taught in AI (Fig. 3).

### Perceived impact of AI on profession

**Fig. 4:**
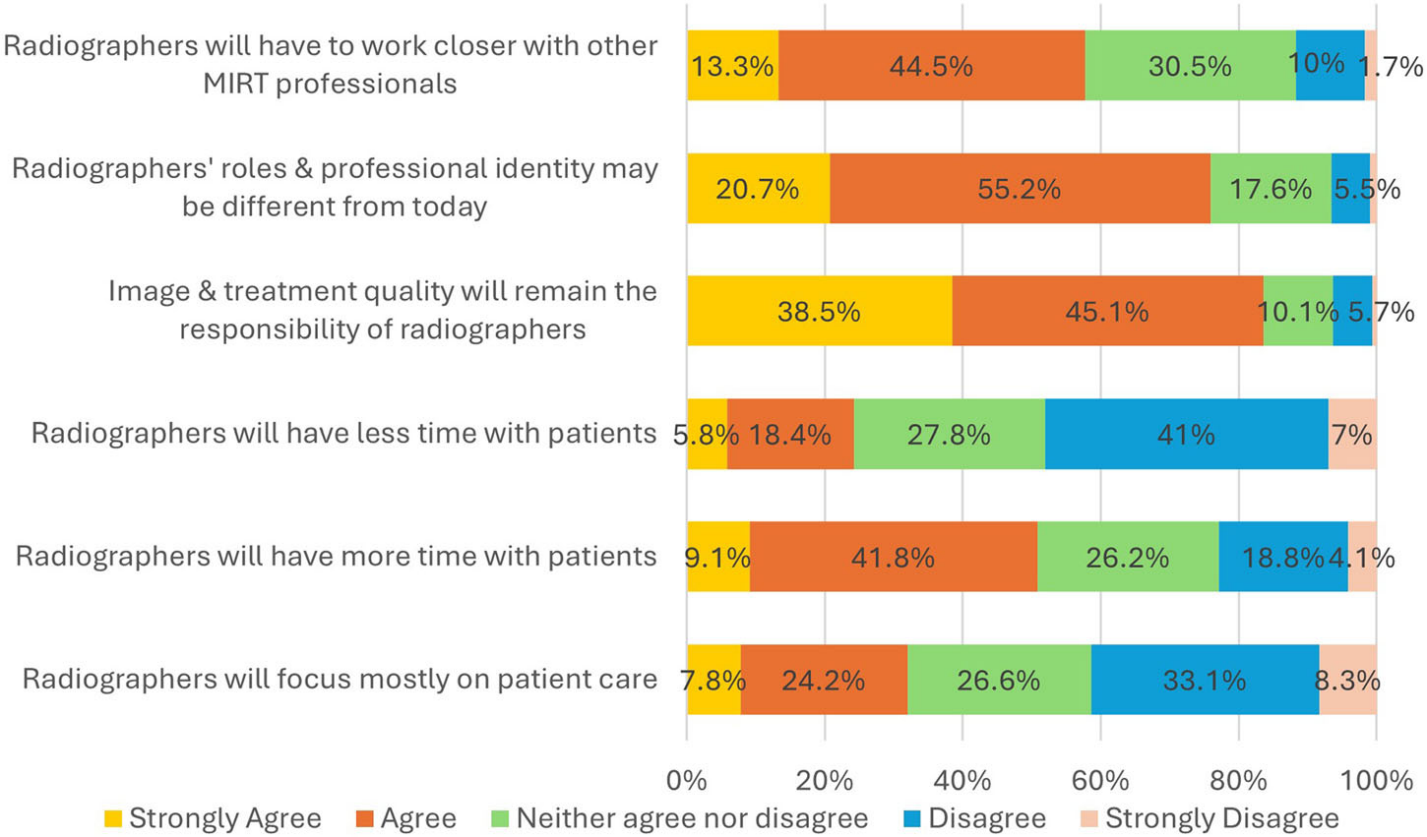
Bar chart summarising the responses for Likert-type questions

Many respondents disagreed (33.1%, *n* = 711) or strongly disagreed (8.3%, *n* = 179) that, with the integration of AI in clinical practice, radiographers will be required to focus mostly on patient care (Fig. 4). Similarly, many disagreed (42.4%, *n* = 891) or strongly disagreed (11.9%, *n* = 250) with the notion that radiographers will be required to focus mostly on technology and less on patient-centred responsibilities. However, they mostly agreed (41.8%, *n* = 866) or strongly agreed (9.1%, *n* = 188) with the opinion that radiographers, with the help of AI, will have more time to spend with patients (Fig. 4).

Over half (51.1%, *n* = 1009) of the respondents thought that the patient-centred care skills of radiographers would remain the same with the advancements of AI, whilst 37.4% (*n* = 739) thought that these skills would increase in importance in the future. Interestingly, 9.8% (*n* = 193) thought that these will decrease. Other responses (1.7%, *n* = 33) mentioned that this would depend on radiographers’ personality or that it was not possible to predict without knowing the full impact that AI may have.

**Fig. 5:**
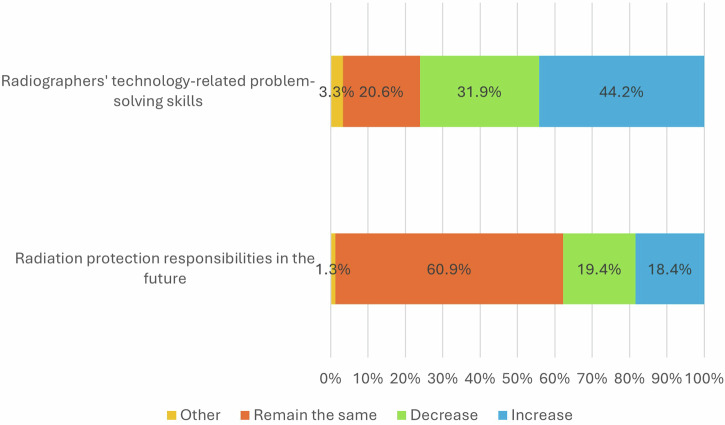
Responses regarding future skills and responsibilities

Most radiographers (83.6%) agreed that despite the advancements of AI, image and radiotherapy treatment quality will remain the responsibility of radiographers, and they will not be replaced by AI in these key tasks. Over half of the respondents (60.9%, *n* = 1195) said that radiation protection responsibilities are likely to remain the same, with the remainder showing a varied opinion (Fig. 5). Despite that, almost 80% of radiographers confirmed they expected their professional roles and identities to change in response to AI integration (Fig. 4).

Over half of the respondents either agreed (44.5%, *n* = 859) or strongly agreed (13.3%, *n* = 256) that radiographers will have to work closer with other professionals in the future.

Many respondents (40.0%, *n* = 882) thought that radiographers’ technology-related problem-solving skills (e.g., quality assurance, or image parameter optimisation) will increase in importance in the future.

Radiographers’ opinions were divided when they were asked about job and career opportunities with the advancement of AI. Approximately one-third (32.3%, *n* = 631) thought that opportunities would increase, 30.1% (*n* = 590) saw a potential decrease, and 32.6% (*n* = 637) of them said these would remain the same. A further 5% (*n* = 98) either were not sure or thought that job and career opportunities would change in different ways.

No significant concerns about role redundancies were reported since the majority of radiographers agreed (33.8%, *n* = 651) or strongly agreed (46.1%, *n* = 886) that AI will only ever assist them and never replace them.

Most of the respondents thought that radiographers will evolve with AI, and roles and professional identity may be quite different from today (Fig. 4). Many of them (44.9%, *n* = 862) agreed or strongly agreed (9.8%, *n* = 189) that radiographers will be more involved in research and development than in their current roles.

### Inferential statistics

Inferential analyses showed that radiographers from EU countries with low digital literacy levels [26] believed more strongly that AI advancements would require closer collaboration with other MIRT professionals (mean score: 3.64) than those from high digital literacy countries (mean score: 3.56; *p*-value: 0.039). Radiographers from countries with low digital literacy levels also thought that AI integration would lead them to focus more on technology and less on patient-related tasks (mean scores: 2.65 vs. 2.47; *p*-value: 0.001). Conversely, radiographers from countries with high digital literacy levels (*n* = 592) believed AI would shift the focus more towards patient care (mean scores: 3.40 vs. 3.30; *p*-value: 0.001) and expected more responsibilities for radiographers towards radiation protection (mean: 2.00 vs. 1.93; *p*-value: 0.027).

Radiographers with some prior AI education believed more strongly that AI would require closer work with other professionals (mean: 3.68 vs. 3.47; *p*-value: < 0.001) and that their roles would evolve significantly (mean: 3.75 vs. 3.61; *p*-value: < 0.001) than those without any AI education. Those with AI education also anticipated more job and career opportunities due to AI (mean: 2.15 vs. 1.89; *p*-value: < 0.001) than those without.

The results also showed that radiographers from countries with EQF6 level education [28] were more confident that they will have to mostly focus on patient care (mean: 2.97 vs. 2.72; *p*-value: < 0.001), spend more time with the patient (mean: 3.38 vs. 3.20; *p*-value: < 0.001) and work closer to patients (mean: 3.28 vs. 3.15; *p*-value: 0.002) compared to those with vocational education. On the contrary, radiographers with vocational-only training were more positive that career opportunities would improve because of AI (mean: 2.14 vs. 1.98; *p*-value: < 0.001).

Some further significant differences were noted between male and female respondents’ opinions (Table 3). It must be noted that further analyses involving non-binary participants were not feasible due to their low prevalence in the sample (0.5%), which does not allow for meaningful comparisons and correlations.

**Table 3:** Opinions on AI with regard to gender identity

Statement	Gender identity male (M) vs. female (F)	*n*	Mean	Std. deviation	*p*-value
With regard to AI advancements, radiographers will need to work closer with the patients	M	691	3.33^+^	0.905	0.001
	F	1230	3.20^+^	0.909	
AI will only ever assist radiographers, never replace them	M	687	4.08^+^	1.027	0.008
	F	1214	4.20^+^	0.982	
With time, AI will ultimately replace radiographers	M	687	2.05^+^	1.066	0.022
	F	1214	1.95^+^	1.049	
Radiographers will evolve with AI, and roles and professional identity may be quite different from today	M	687	3.71*	0.644	0.023
	F	1213	3.67^+^	0.611	
With the integration of AI in clinical practice, radiographers will be required to focus mostly on patient care (consent, positioning, cannulation) and be less involved in technology	M	746	2.82^+^	1.111	0.012
	F	1372	2.95^+^	1.092	
Despite the advancement of AI, image quality and treatment quality will remain the responsibility of the radiographers	M	709	4.07^+^	0.907	0.001
	F	1295	4.20^+^	0.833	
With regard to AI advancements, radiographer technology-related problem-solving skills (e.g., quality assurance or optimisation of imaging/treatment parameters to suit patient anatomy and pathology) will improve	M	682	2.20*	0.872	0.008
	F	1223	2.09*	0.882	
With regard to AI advancements, radiographer patient-centred skills (e.g., adaptive techniques and communication to address patient needs) will improve	M	697	2.31*	0.682	0.005
	F	1250	2.22*	0.686	
With regard to AI advancements, radiographer job and career opportunities will improve	M	668	2.14*	0.798	0.000
	F	1171	1.95*	0.812	

(M) signifies male respondents and (F) signifies female respondents. Their mean, standard deviation and *p* value of their difference (M vs F) are also mentioned in this table. This table includes only the differences that were statistically significant

* Analysed on a 3-point scale with scores ranging from 1 to 3, where 1 = decrease, 2 = remain the same, and 3 = increase

^+^ Analysed on a 5-point scale with scores ranging from 1 to 5, where 1 = strongly disagree, 2 = disagree, 3 = neither agree nor disagree, 4 = agree, and 5 = strongly agree

Those with more AI experience were more likely to disagree with the notion that AI would shift the focus to technology and away from patient-related responsibilities (correlation: −0.063; *p*-value: 0.004). They were also more likely to believe that radiographers would maintain responsibility for image and treatment quality (correlation: 0.053; *p*-value: 0.017). Additionally, they tended to disagree that AI would take over this responsibility (correlation: −0.100; *p*-value: < 0.001). More AI experience was also associated with the belief that AI will improve job and career opportunities (correlation: 0.152; *p*-value: < 0.001). Respondents with greater AI experience were more likely to believe that AI would assist rather than replace radiographers (correlation: 0.047; *p*-value: 0.038).

Regarding the age of respondents, younger radiographers (18–35 years old) expressed greater fears of being replaced by AI, compared to the 36+ years age group (mean: 2.10 vs. 1.90; *p*-value: 0.005). Younger radiographers more strongly believed that AI would increase their research roles (mean: 3.51 vs. 3.41; *p*-value: 0.003).

**Fig. 6:**
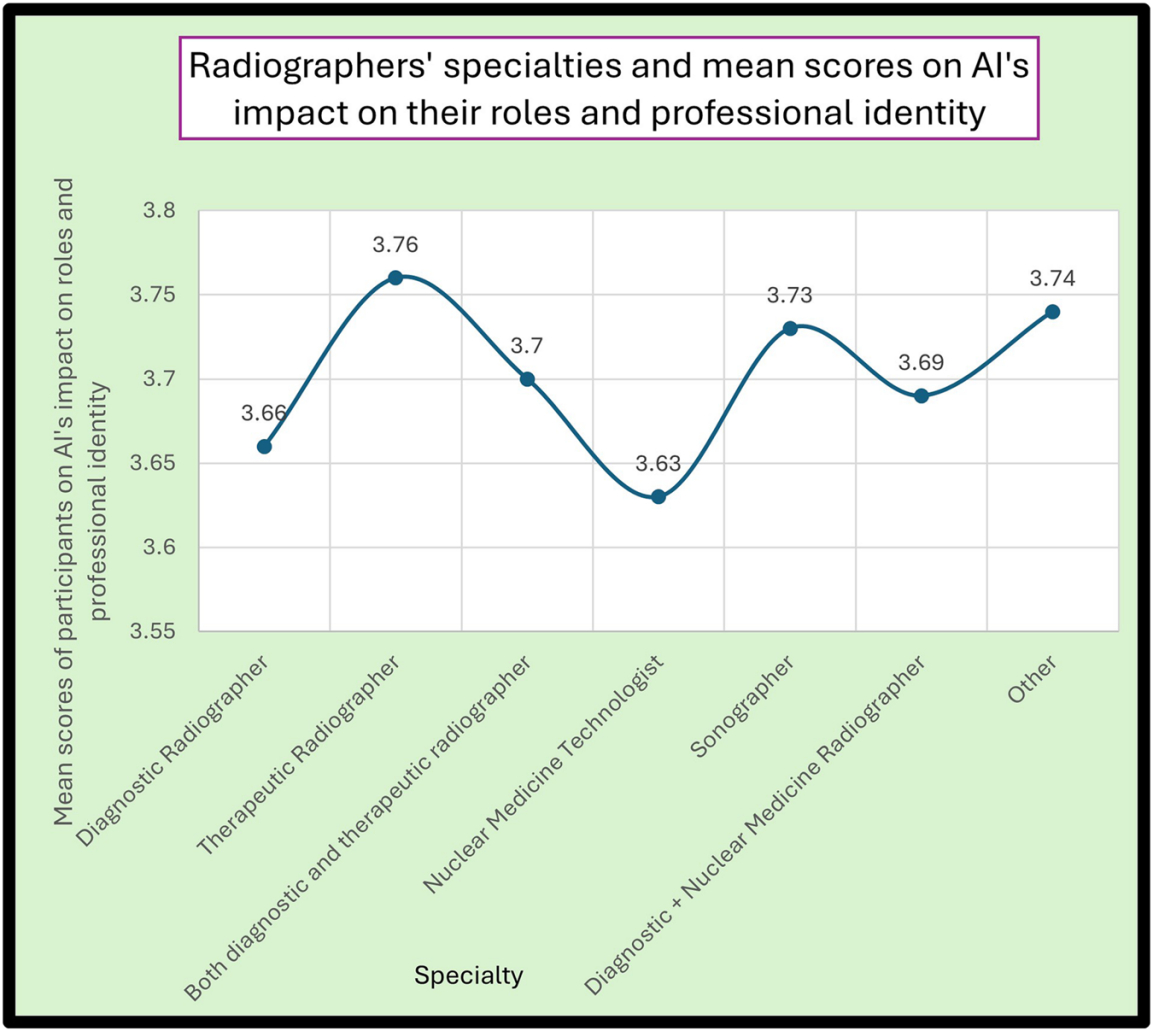
Radiographers’ specialties and mean scores on AI’s impact on their roles and professional identity

Finally, with respect to radiographers’ roles and professional identity evolving with AI, therapeutic radiographers and sonographers were more positive compared to other specialties within the profession (mean: 3.76 and 3.73, respectively). Conversely, nuclear medicine radiographers were less positive about this (Fig. 6).

## Discussion

This is, to our knowledge, the largest survey of its kind in the MIRT professions in Europe. Previous research has shown that radiographers will play a pivotal role as a professional group in the adoption of AI in clinical practice [3, 13, 29, 30]. Rigorous AI governance frameworks, staff education/training, collaborative research, effective leadership, and patient and public involvement are needed to ensure a safe and successful implementation of AI in MIRT [31].

The findings of this study show a promising increase in the AI awareness levels of radiographers (Fig. 2), compared to previous studies, which have reported generally lower levels of AI awareness [32–34]. This can be attributed to the systematic work of both universities and professional bodies to promote AI knowledge among radiography professionals [35, 36]. Despite an increase in AI awareness, AI education has not followed a similar pattern (Fig. 3). Increasing awareness of the impact of AI on radiography practice combined with a simultaneous lack of AI education and expert skills creates a mismatch of knowledge and skills and, subsequently, distrust of new technology. Low trust levels in a potentially highly effective technology could lead to its rejection or suboptimal use, resulting in high costs and inefficiencies. Conversely, high trust levels in potentially ineffective AI applications could lead to incongruous over-reliance and misuse, which may result in patient safety breaches and other undesirable outcomes [37]. Despite some progress in recent years, the responses in this survey also indicate that most radiographers reported low technical self-efficacy in using AI. Self-efficacy is a person’s belief in their ability to take action to achieve specific goals [38]. Low technical self-efficacy can lead to avoidance behaviour, reduced work effort, and outright resistance [39].

Our results demonstrate that despite increasing awareness, the available educational provisions are not reaching many radiography practitioners. Education is vital to bridge the gap between increasing awareness and lack of AI-specific knowledge [40]. While European radiographers have already started to invest in AI education by developing new courses or webinars (led by universities, professional bodies, and congresses across Europe), they need to do this at pace and scale, to ensure competency in their roles [13, 41]. This is vital given the latest requirements of the EU AI Act for digital literacy of all healthcare professionals [42]. In the UK, radiographer registrations require distinct digital aptitudes, delivered by accredited higher education institutions [43]. Benchmarking documents released by the International Society of Radiographers and Radiological Technologists (ISRRT), EFRS, and the Society and College of Radiographers with regard to EQF [44] and AI [45] should be proportionately updated [46].

The results show an overall optimistic workforce (Fig. 6), with men slightly more enthused about the development of technological skills and women about the honing of patient-centred care skills (Table 3), which has been also confirmed in previous research [47]. Younger professionals seemed more concerned about being replaced, which might relate to their chosen sources of information, like social media, where unfounded scaremongering about AI might be more common. In addition, the results of this study demonstrated that certain radiography disciplines, like sonographers, nuclear medicine technologists and therapeutic radiographers, were more positive towards AI compared to the rest of radiography specialties. Although previous studies exist regarding the perceptions of radiographers about AI, the variations in career advancement, career opportunities, professional recognition, societal recognition, and establishment of professional identity across different radiography disciplines may contribute to these results [48–50].

Previous research has confirmed the relationship between AI knowledge and fear of replacement among other MIRT professionals [9]. Our results also suggest that higher levels of digital literacy, higher levels of radiography education (EQF level 6 and above), prior AI education, and AI experience enabled more optimistic views from participants about the future of careers in the AI era. Furthermore, they cultivate a more humanistic and person-centred approach to healthcare for clinical practitioners. To enable adoption, it is important that user participation in the development and customisation of AI applications and operational experience enhances acceptance of AI through the cognitive path (i.e., enhancing AI self-efficacy) and the affective path (i.e., lowering AI anxiety) [51]. The findings of this study suggest that digital literacy mediates the motivation for the adoption of AI. The high digital literacy subgroup focused on patient-centred care, while the low digital literacy group saw their involvement in addressing the expected technology complexity as a relative advantage for career advancement and trigger factor for adoption.

Overall, the findings of this study show that European radiographers express positive opinions towards the adoption of AI (Fig. 4), believing that AI will mostly assist them in their new roles and responsibilities, without expressing fears of losing their jobs. These findings align with results from previous research conducted among radiologists and radiology residents, with overall positive attitude towards AI [9]. The respondents of our study underline the need to work closely with other professionals to enhance patient experience and patient outcomes. Radiographers fully appreciate the duality of their roles between technology and patient care, and they value working with patients (Fig. 4).

### Future research

Future research should be conducted to shed light on the real-world challenges that radiographers face when using AI solutions in clinical practice; this will allow radiography academics to customise AI education/training both at undergraduate and postgraduate levels to meet the needs and expectations of future professionals and future career opportunities, as shaped by AI. Future radiography AI education should include both theoretical knowledge and hands-on training, so students can gain a holistic experience in the use of AI solutions in clinical practice. Interprofessional faculty approaches should be employed by higher education institutions when designing such AI courses for radiographers [41]. Clinical practice and radiography training should take into account the recent governance requirements [31] as stipulated by the EU AI act [42] and the Health and Care Professions Council (HCPC) [43] and other regulatory bodies nationally and internationally, to enhance AI transparency, digital literacy, and data privacy for the benefits of patients and professionals.

### Limitations

First, this was an online survey advertised on social media and related networks, which might have inadvertently excluded from participation those radiographers with no, or limited access to Internet and social media. Second, the use of radiographers as translators might have resulted in some linguistic inaccuracies; however, because they were professionals with knowledge of the socio-economic context, background and history of the radiography profession in their country, we believe they were ideally suited to perform not just the translation but also the required interpretation of what was written onto the survey. Finally, self-selection bias might have occurred in this study, due to the voluntary nature of participant recruitment.

## Conclusion

With radiography being one of the most digitally mature professions in healthcare, it is vital to understand the impact on the workforce, who will deliver this digital transformation. European radiographers have overall optimistic views about the use of AI in healthcare. Some statistically significant differences with gender, level of radiography education, and digital literacy were observed. Despite increasing awareness of AI in their practice, AI education currently lags for European radiographers, and this should be acutely addressed at the scale and pace required to keep up with current technological developments. This was also demonstrated from our findings that AI education, digital literacy and prior AI experience show a strong correlation with optimistic views about AI integration and the future of radiographer jobs and career prospects. Interprofessional collaboration is essential not only for the seamless integration of AI into clinical practice but also for fostering mutual support among professionals, ultimately benefiting patients.

## Supplementary information

ELECTRONIC SUPPLEMENTARY MATERIAL

## Data Availability

The data is not publicly available due to privacy and ethical restrictions.

## Abbreviations

EFRS: European Federation of Radiographer Societies
EQF: European Qualification Framework
MIRT: Medical imaging and radiotherapy
UKIO: UK Imaging & Oncology Congress
