# Supplementary material for: R-AI-diographers: a European survey on perceived impact of AI on professional identity, careers, and radiographers’ roles
Source: Insights Imaging. 2025 Feb 17;16:43. doi: 10.1186/s13244-025-01918-6 (PMC11832980; doi:10.1186/s13244-025-01918-6)
Supplement: Supplementary file 1 — ELECTRONIC SUPPLEMENTARY MATERIAL [file 13244_2025_1918_MOESM1_ESM.pdf]

R-AI-diographers: a European survey on perceived impact of AI on professional identity, careers, and radiographers' roles

ELECTRONIC SUPPLEMENTARY MATERIAL

Summary of survey key questions and structure

**Demographics**	Age
	Gender identity
	Country of practice
	Experience in years
	Current role
	Radiography specialty
	Highest academic qualification
	Type of clinical setting
**AI knowledge**	Knowledge of AI
	Forms of AI education/training
	Experience in using AI
**Clinical practice**	Focus on patient care
	Focus on technology
	Time spent with patients
	Working with other professionals
**Responsibilities**	Image and treatment quality
	Radiation protection
**Skills**	Patient-centred skills
	Problem-solving skills
**Roles and identity**	Job and career opportunities
	Fears of replacement
	Identity changes
	Involvement in research
